# P-1884. Evaluation of Outpatient Parenteral Antimicrobial Therapy Practices within a Large Healthcare System

**DOI:** 10.1093/ofid/ofae631.2045

**Published:** 2025-01-29

**Authors:** Christina Beran, Chungyun Kim, Emily Wings, Andrea Pallotta, Nabin K Shrestha

**Affiliations:** Cleveland Clinic, Cleveland, Ohio; Cleveland Clinic, Cleveland, Ohio; Cleveland Clinic, Cleveland, Ohio; Cleveland Clinic, Cleveland, Ohio; Cleveland Clinic, Cleveland, Ohio

## Abstract

**Background:**

The practice of outpatient parenteral antimicrobial therapy (OPAT) has challenges such as reduced control and monitoring in the outpatient setting. Lack of availability of OPAT lab monitoring is associated with poorer outcomes. This study sought to describe the operations and practices of an OPAT program within a large health system to identify clinical and operational opportunities for workflow improvement.

Laboratory monitoring completion over proportion of OPAT courses

**Figure:**
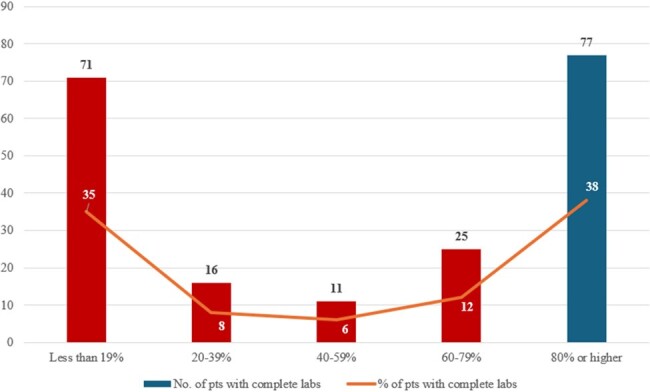

**Methods:**

This was a retrospective cohort study of patients receiving OPAT in a large healthcare system from January 1, 2022 to December 31, 2022. After excluding OPAT courses initiated in the outpatient setting and courses less than 7 days long, 200 OPAT courses for patients greater than 18 years of age were selected, using a random sample generator, from the Cleveland Clinic OPAT registry. Events were followed until the OPAT end date or 30 days, whichever occurred sooner. The relationship between lab monitoring completion within 24 hours of scheduled date and detection of clinically significant lab abnormalities (CSLAs) was examined using linear regression. Secondary outcomes included the rate of emergency department (ED) visits and readmissions.

Cumulative incidence of ED visits by target lab monitoring level

**Figure:**
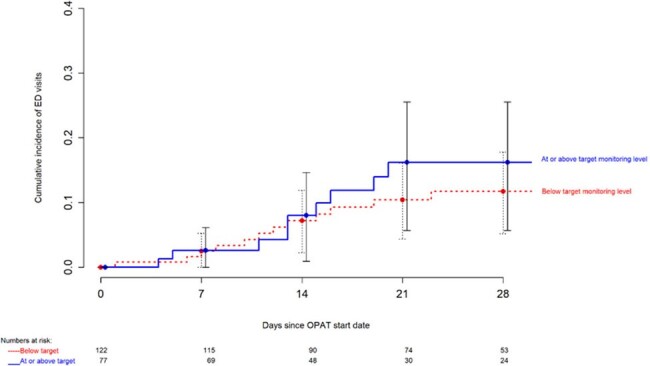

**Results:**

The mean age (SD) of included patients was 63 years (27) and 64% were male. Fewer than half the OPAT courses had 80% or higher lab monitoring completion (Figure 1). Detected CSLAs are shown in Table 1. In a linear regression model adjusted for high-risk medications, the number of CSLAs detected per OPAT course increased by 0.003 (95% C.I. 0.002 – 0.004) per unit increase in percentage lab monitoring completion (p < 0.001). ED visit within 30 days was 11% (Figure 2) and readmission within 30 days was 23% (Figure 3). Reasons for readmission included non-OPAT-related (53%, n=24 of 45), infection-related (40%, n=18 of 45), and adverse OPAT drug effects (9%, n=4 of 45).

Cumulative incidence of readmissions by target lab monitoring level

**Figure:**
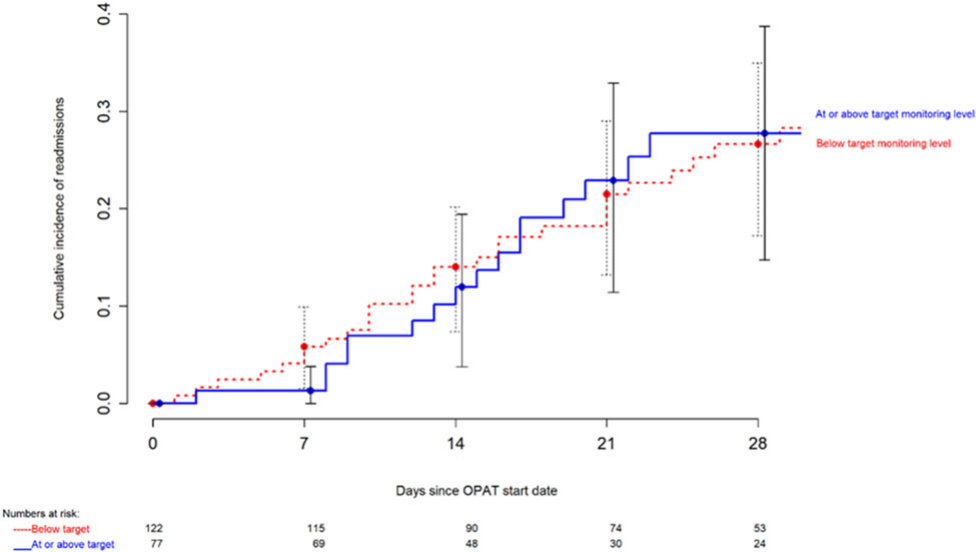

**Conclusion:**

This study showed a substantial gap in lab completion and a significant association between lab monitoring completion and detection of CSLAs, thereby demonstrating clinical and operational opportunity for OPAT workflow improvement with monitoring and follow-up actions. A multidisciplinary OPAT team with clearly defined roles may enhance safety, effectiveness, and efficiency within the program.

Detection of Clinically Significant Lab Abnormalities

**Figure:**
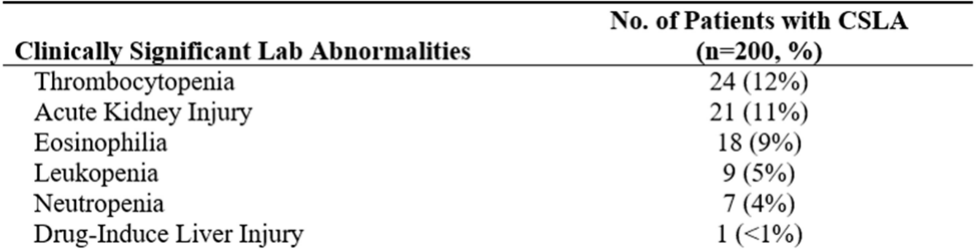

**Disclosures:**

Andrea Pallotta, PharmD, Astra Zeneca: Advisor/Consultant|Viiv: Advisor/Consultant

